# Performance of T-Track^®^ TB, a Novel Dual Marker RT-qPCR-Based Whole-Blood Test for Improved Detection of Active Tuberculosis

**DOI:** 10.3390/diagnostics13040758

**Published:** 2023-02-16

**Authors:** Johannes P. Meier, Selina Möbus, Florian Heigl, Alexandra Asbach-Nitzsche, Hans Helmut Niller, Annelie Plentz, Korkut Avsar, Marion Heiß-Neumann, Bernhard Schaaf, Uwe Cassens, Bernd Seese, Daniel Teschner, Sabin Handzhiev, Uwe Graf, Christoph Lübbert, Monika Steinmaurer, Konstantina Kontogianni, Christoph Berg, Andreas Maieron, Stefan H. Blaas, Ralf Wagner, Ludwig Deml, Sascha Barabas

**Affiliations:** 1 Mikrogen GmbH, 82061 Neuried, Germany; 2 Lophius Biosciences GmbH, 93053 Regensburg, Germany; 3 Institute for Medical Microbiology and Hygiene, University of Regensburg, 93053 Regensburg, Germany; 4 Lungenärzte am Rundfunkplatz, 80335 Munich, Germany; 5 Department of Tuberculosis/Infectious Lung Disease, Asklepios Lungenklinik Gauting, 82131 Gauting, Germany; 6 Medizinische Klinik Nord, Klinikum Dortmund gGmbH, 44145 Dortmund, Germany; 7 Institut für Transfusionsmedizin, Laboratoriumsmedizin und Medizinische Mikrobiologie, Klinikum Dortmund gGmbH, 44137 Dortmund, Germany; 8 Fachklinik für Pneumologie, Thoraxchirurgie, Rehabilitation, Schlaf- und Beatmungsmedizin, 97702 Münnerstadt, Germany; 9 Department of Haematology and Medical Oncology, University Medical Centre of the Johannes Gutenberg University, 55131 Mainz, Germany; 10 Department of Internal Medicine II, University Hospital Würzburg, 97080 Würzburg, Germany; 11 Karl Landsteiner Privatuniversität für Gesundheitswissenschaften, Universitätsklinikum Krems, 3500 Krems an der Donau, Austria; 12 Innere Medizin IV, Klinikum Chemnitz gGmbH, 09116 Chemnitz, Germany; 13 Department of Infectious Diseases and Tropical Medicine, Klinikum St. Georg gGmbH, 04129 Leipzig, Germany; 14 Department of Medicine I, Division of Infectious Diseases and Tropical Medicine, Leipzig University Hospital, 04103 Leipzig, Germany; 15 Klinikum Wels-Grieskirchen GmbH, 4600 Wels, Austria; 16 Department for Pneumology and Critical Care Medicine, Thoraxklinik at University of Heidelberg, 69126 Heidelberg, Germany; 17 Translational Lung Research Center Heidelberg, German Center for Lung Research, 69120 Heidelberg, Germany; 18 Department of Internal Medicine I, Tübingen University Clinical Centre, 72016 Tübingen, Germany; 19 Internal Medicine 2, Gastroenterology and Hepatology and Rheumatology, Karl Landsteiner University of Health Sciences, University Hospital of St. Pölten, 3100 Sankt Pölten, Austria; 20 Center for Pneumology, Donaustauf Hospital, 93093 Donaustauf, Germany; 21 Institute for Clinical Microbiology and Hygiene, University Hospital Regensburg, 93053 Regensburg, Germany

**Keywords:** tuberculosis, TB, active TB, infection detection, T-Track^®^ TB, QuantiFERON^®^-TB Gold Plus, mRNA, RT-qPCR, CXCL10, IFNG

## Abstract

Tuberculosis (TB) is one of the leading causes of death by an infectious disease. It remains a major health burden worldwide, in part due to misdiagnosis. Therefore, improved diagnostic tests allowing the faster and more reliable diagnosis of patients with active TB are urgently needed. This prospective study examined the performance of the new molecular whole-blood test T-Track^®^ TB, which relies on the combined evaluation of *IFNG* and *CXCL10* mRNA levels, and compared it to that of the QuantiFERON^®^-TB Gold Plus (QFT-Plus) enzyme-linked immunosorbent assay (ELISA). Diagnostic accuracy and agreement analyses were conducted on the whole blood of 181 active TB patients and 163 non-TB controls. T-Track^®^ TB presented sensitivity of 94.9% and specificity of 93.8% for the detection of active TB vs. non-TB controls. In comparison, the QFT-Plus ELISA showed sensitivity of 84.3%. The sensitivity of T-Track^®^ TB was significantly higher (*p* < 0.001) than that of QFT-Plus. The overall agreement of T-Track^®^ TB with QFT-Plus to diagnose active TB was 87.9%. Out of 21 samples with discordant results, 19 were correctly classified by T-Track^®^ TB while misclassified by QFT-Plus (T-Track^®^ TB-positive/QFT-Plus-negative), and two samples were misclassified by T-Track^®^ TB while correctly classified by QFT-Plus (T-Track^®^ TB-negative/QFT-Plus-positive). Our results demonstrate the excellent performance of the T-Track^®^ TB molecular assay and its suitability to accurately detect TB infection and discriminate active TB patients from non-infected controls.

## 1. Introduction

Tuberculosis (TB), caused by *Mycobacterium tuberculosis* (*Mtb*) bacteria, is a global health problem, with 1.6 million death cases estimated in 2021 [1,2]. Moreover, the World Health Organization (WHO) estimated that 1.8 billion people are latently infected with *Mtb* and, thus, are at potential risk of developing active TB disease during their lifetime [2]. Due to the lack of effective vaccines, the early and reliable diagnosis and treatment of TB patients represent the most effective approach to contain the pandemic. According to the WHO, an estimated 66 million lives have already been saved by this strategy between 2000 and 2020 [1].

Various microbiological, radiological, and immunological tests are available to diagnose TB. Microbiological tests such as smear microscopy, *Mtb* culture, and nucleic acid amplification tests (such as GeneXpert^®^ MTB/RIF assay, Cepheid) are the most widely used to diagnose active TB. In contrast, immune-based diagnostic tools such as the Tuberculin Skin Test (TST) and Interferon Gamma Release Assays (IGRAs) are primarily recommended for the detection of latent TB infection [3,4,5]. The TST has limited specificity in detecting *Mtb* infection due to false positive results in individuals with either prior immunization with the Bacillus Calmette–Guérin (BCG) vaccine or exposure to non-tuberculous mycobacteria (NTM) [6]. In contrast, currently available commercial IGRAs such as T-SPOT.TB (Oxford Immunotec), an enzyme-linked immunosorbent spot (ELISpot)-based assay quantifying IFN-γ spot-forming cells, and Quantiferon^®^-TB Gold Plus (Qiagen), an enzyme-linked immunosorbent assay (ELISA)-based method measuring the IFN-γ concentration in the serum of blood stimulated with *Mtb* antigens, show improved sensitivity and specificity in latent TB infection, particularly in BCG-vaccinated individuals [3,7,8,9]. However, TST and IGRAs have a relatively high false negative rate in patients with active TB [7,8,10,11], particularly in young children and immunocompromised individuals [12,13,14]. Therefore, a high clinical need remains for improved tests for the early and robust diagnosis of active tuberculosis, particularly in patients with extrapulmonary TB, smear-negative status, immunocompromised patients, and children.

Several promising approaches have been discussed to improve the performance of existing IGRAs. Among them are the use of superior readout procedures and the measurement of additional analytes. In this context, molecular methods such as quantitative reverse transcription–polymerase chain reaction (RT-qPCR) have been proposed as a more sensitive approach to measuring analytes and facilitating the simultaneous detection of multiple markers [15,16]. A further strategy is to consider the combination of analytes to improve TB diagnosis. Of particular interest is the chemokine CXCL10, which is produced in large amounts by antigen-presenting cells, particularly monocytes, in response to IFN-γ signaling, thereby amplifying the IFN-γ response of T cells to antigenic stimulation [17,18,19]. CXCL10 has been the focus of attention as a versatile TB biomarker for quite some time [20,21,22,23,24,25,26]. CXCL10 is most consistently expressed in response to TB antigen challenge, rendering it the most promising alternative marker to IFN-γ [21,23,24,27] and a suitable means of improving the performance of IGRAs [20]. CXCL10 is elevated in adult patients with active TB compared to unexposed subjects, and produced at high levels following stimulation of the whole blood of TB patients by TB-specific antigens [17]. Several studies also reported that CXCL10 might represent a robust marker in diagnosing TB in young children, HIV-infected individuals with low CD4 cell counts, and patients with inflammatory rheumatic diseases [18,21,28,29,30,31,32].

T-Track^®^ TB (Mikrogen GmbH, Neuried, Germany) is a novel Conformité Européene (CE)-marked in vitro diagnostic RT-qPCR infection detection test based on the combined assessment of the relative mRNA level of both *IFNG* and *CXCL10* in specifically restimulated vs. non-stimulated whole-blood samples, as indirect biomarkers of *Mtb* infection. This prospective study aimed to investigate the performance of T-Track^®^ TB in comparison to the currently widely used QuantiFERON^®^-TB Gold Plus (QFT-Plus) assay. Due to the lack of a gold-standard test for latent *Mtb* infection, a case–control study comparing patients with confirmed active TB disease to non-infected controls was conducted. Our results demonstrate the excellent performance of T-Track^®^ TB and support its suitability for the diagnosis suspected active TB.

## 2. Materials and Methods

### 2.1. Study Design and Participants

A total of 541 individuals, including 273 patients with suspected active tuberculosis and 268 non-infected controls, were enrolled between April 2019 and June 2022 from 14 healthcare centers in Germany and Austria (Table S1). Patients with active TB were diagnosed either by a positive *Mtb* culture or positive microscopy and PCR results. Non-infected controls were confirmed to have negative Quantiferon^®^-TB Gold Plus (hereafter referred to as QFT-Plus) (Qiagen, Hilden, Germany) results. Subjects below 18 years of age, pregnant women, as well as individuals with an inability or unwillingness to provide informed consent, known to be infected with human immunodeficiency virus (HIV), hepatitis B virus (HBV), or hepatitis C virus (HCV), who received, for more than seven days, medication for the treatment of active TB, and patients under immunosuppressive therapy (including conventional agents such as azathioprine or everolimus, biologics such as infliximab or golimumab monoclonal antibodies, or >10 mg steroid per day), were excluded from enrolment. Non-infected controls with a previous risk of TB exposure were also excluded. The risk of TB exposure was assessed by a questionnaire survey about previous TB history, including personal contact with TB patients, history of stay > 3 months in high-TB-incidence countries (e.g., South Africa, China, India, Russia), and the existence of symptoms suggestive of a TB infection at the time of enrolment.

The study was approved by the Ethics Committee of the University of Regensburg, Germany (approval number 17-571-122, dated 17 July 2017) and subsequently by the Ethics Committees of all other participating healthcare centers. Written consent was obtained from all individuals enrolled in the study.

### 2.2. Sample Collection 

Blood samples (15 mL) were collected in lithium–heparin collection tubes (Sarstedt S-Monovette^®^ 7.5 mL LH, Nümbrecht, Germany). Samples collected at different locations (Table S1) were transported per courier at room temperature (18–25 °C) within 8 h of withdrawal to one of the two measuring laboratories (Mikrogen GmbH, Regensburg, Germany; Institute for Transfusion Medicine, Laboratory Medicine and Medical Microbiology, Medical Center Dortmund, Dortmund, Germany). Upon arrival, blood samples were immediately aliquoted to perform T-Track^®^ TB and QFT-Plus assays simultaneously on the same sample materials.

### 2.3. T-Track^®^ TB Assay

**Figure 1:**
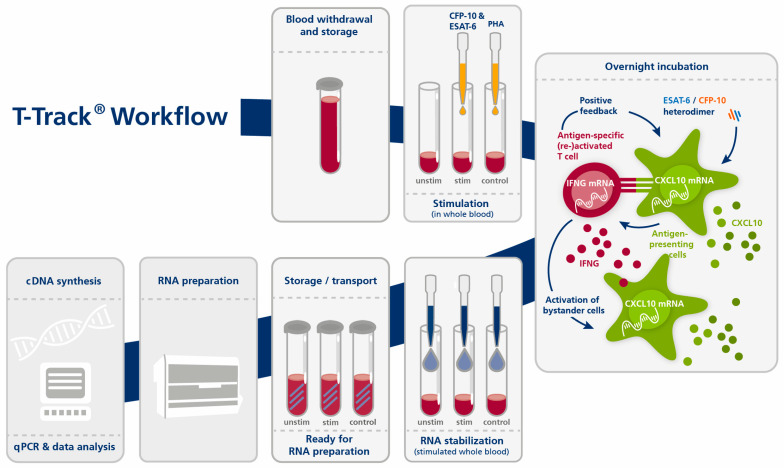
Principle and workflow of the T-Track^®^ TB assay. Abbreviations: cDNA, complementary DNA; control, stimulation control (PHA-stimulated); ESAT-6, Early Secreted Antigenic Target 6 kDa (TB antigen); CFP-10, Culture Filtrate Protein 10 (TB antigen); CXCL10, C-X-C motif chemokine ligand 10 (biomarker); IFNG, interferon gamma (biomarker); PHA, phytohemagglutinin; qPCR, quantitative PCR; stim, stimulated (with TB antigens); unstim, unstimulated negative control.

T-Track^®^ TB consists of the T-Track^®^ TB Stimulation Kit (Cat. No. 11001004, Mikrogen GmbH, Neuried, Germany) and the T-Track^®^ TB Quant PCR Kit (Cat. No. 11001005, Mikrogen GmbH). T-Track^®^ TB is intended as an aid in the diagnosis of *Mtb* infection, including active TB disease. T-Track^®^ TB was performed according to the manufacturer’s instructions (Figure 1). Briefly, each 1 mL heparinized blood was transferred into three tubes and stimulated with the TB antigen or with phytohemagglutinin (PHA) as a stimulation control. The third tube served as a non-stimulated negative control. 

The TB antigen used in this study is a recombinant ESAT-6/CFP-10 heterodimer protein produced in BL21 (DE3) *E. coli* upon cloning of the respective genes into pET21a, in fusion with a C-terminal His-tag. ESAT-6 was purified on a HisTrap column (HisTrap FF crude 5 mL, GE17-5286-01, GE Healthcare/Merck, Darmstadt, Germany) under denaturing conditions (8 M urea) and refolded on the column by a decreasing urea gradient. CFP-10 was purified on a HisTrap column (HisTrap FF crude 5 mL, GE17-5286-01, GE Healthcare/Merck, Darmstadt, Germany) under standard conditions. After elution from the HisTrap column with imidazole, ESAT-6 and CFP-10 proteins were further purified on a HiTrap anion exchange column (HiTrap Q HP, GE17-1154-01, GE Healthcare/Merck) and by size exclusion chromatography (HiPrep 26/60 Sephacryl S-100HR, GE17-1194-01, GE Healthcare/Merck, Darmstadt, Germany). LPS content was verified to be <0.1 endotoxin units (EU)/mg using the EndoSafe™ Kinetic Chromogenic LAL Assay (R17100K, Charles River, Wilmington, MA, USA). The TB antigen was prepared by mixing equal amounts (mass) of purified ESAT-6 and CFP-10 proteins to generate a stable 1:1 heterodimeric complex, as previously reported [33,34].

After adding the stimulants, blood samples were incubated overnight (15 to 23 h) in a 37 °C incubator. RNA was then stabilized by adding T-Track^®^ RNA Stabilizer to each sample. RNA was extracted using a MagNA Pure 96 device and the MagNA Pure 96 Cellular RNA Large Volume Kit (Roche, Penzberg, Germany; Cat. No. 05467535001) and stored at −80 °C until further use. RT-qPCR was performed with the T-Track^®^ TB Quant PCR Kit using the extracted RNA as a template, in accordance with the manufacturer’s instructions. RT-qPCR was run on a QuantStudio 5 real-time PCR system (ThermoFisher Scientific, Waltham, MA, USA) using the following parameters: 10 min at 50 °C and 30 s at 95 °C for the reverse transcription step, followed by 38 cycles of 30 s at 95 °C and 30 s at 60 °C. The *IFNG*, *CXCL10*, and *RPLP0* genes were analyzed in parallel. All samples were analyzed using the T-Track^®^ TB Calculator Version v01.01 (Mikrogen GmbH). The increased expression of IFNG/CXCL10 in the specifically stimulated vs. non-stimulated blood sample is an indirect marker for the presence of *Mtb*-specific activated and memory T cells and indicates previous contact with TB pathogens.

The T-Track^®^ TB assay provides a primary (semi-)quantitative measuring result (the relative amount of RNA [E^(−Ct)] as calculated from the RT-qPCR Ct value) that is transferred into a qualitative test result (“negative” or “positive”) by the T-Track^®^ TB Calculator. This software assesses the validity of the results and classifies samples as “positive” (*Mtb*-reactive T cells were detected), “negative” (*Mtb*-reactive T cells were not detected), “inconclusive”, or “invalid”. 

Briefly, if the validity rules (the measured Ct values of the samples and controls must be within the defined acceptance range for all measured markers) are met, the Ct values for *IFNG*, *CXCL10*, and *RPLP0* are corrected for their respective amplification efficiencies. The *IFNG* and *CXCL10* values (classification markers) are then normalized to the values of *RPLP0* (reference marker), and the fold change (FC) values for *IFNG* and *CXCL10* are calculated (ratio between stimulated and unstimulated states). The FC values are log2-transformed and used to generate a score (using proprietary algorithms) that allows the qualitative classification of each blood sample. The assay classification threshold was established by binary logistic regression using samples of 90 TB-infected patients and 61 non-TB controls. The classification threshold can be plotted as a line on a scatter plot with log2-transformed *CXCL10* FC values on the x-axis and log2-transformed *IFNG* FC values on the y-axis. Values below the threshold line are classified as negative, values above as positive.

Distance values were calculated as the orthogonal distances of the data points (log2-transformed *CXCL10* and log2-transformed *IFNG* FC values) in this scatter plot to the threshold line. Distances of points below the threshold were given a negative sign. Classification based on the threshold and calculation of the distance is only possible if both markers are used for the analysis. In rare cases where only one marker was valid, classification was performed based on the analysis of a single marker only. Classification thresholds for single-marker (IFNG or CXCL10) analysis were determined using a cohort of 90 TB-infected patients and 77 (IFNG) or 61 (CXCL10) non-TB controls. Upper and lower thresholds were defined for positivity and negativity, respectively. Single-marker FC values between the upper and lower thresholds were defined as inconclusive. Optimal upper and lower thresholds were established by minimizing the number of false negative (FN), false positive (FP), and inconclusive results using a proprietary score formula. 

In the event that the validity rules were not met (the measured Ct values of the samples and controls were not within the defined acceptance range for all measured markers), the test was classified as invalid. 

### 2.4. QuantiFeron^®^-TB Gold Plus Assay

QuantiFeron^®^-TB Gold Plus (QFT-Plus) (Qiagen, Hilden, Germany) was performed according to the manufacturer’s instructions. Briefly, each 1 mL heparinized blood was transferred into four tubes (Nil, TB1, TB2, and Mitogen). Tubes were repeatedly inverted and subsequently incubated for 16–24 h at 37 °C. The four tubes were then centrifuged for 15 min at 2500× *g*, and the separated plasma was stored at −20 °C until ELISA testing. The QFT-Plus ELISA was performed on an RT-6100 microplate reader (Rayto Life and Analytical Sciences, Shenzhen, China). The results were interpreted by applying the QFT-TB Analysis Software provided by Qiagen (www.quantiferon.com [accessed on 15 February 2023]). IFN-γ levels (IU/mL) in TB1, TB2, and Mitogen tubes were adjusted after subtracting Nil tube levels and were expressed as TB1-Nil, TB2-Nil, and Mitogen-Nil, respectively. When the IFN-γ values of TB1-Nil or TB2-Nil were ≥ 0.35 IU/mL and ≥ 25% of the Nil value, the result was considered positive, and the reverse was true for negative results. If the IFN-γ value of the Nil tube was > 8.0 IU/mL or that of the mitogen tube was < 0.5 IU/mL, the result was considered indeterminate (also labelled as “inconclusive” in this study).

### 2.5. Statistical Analysis

Data were analyzed using Microsoft Excel for Microsoft 365 (Microsoft Corporation, Redmond, WA, USA) and GraphPad Prism 9 (Dotmatics, Boston, MA, USA). 

In the case of multiple samples per patient, only the first available sample (chronologically) was included in the analysis. 

Sensitivity was defined as the ratio of the number of true positive (TP) assessments to the number of all positive assessments (TP + FN). Specificity was defined as the ratio of the number of true negative (TN) assessments to the number of all negative assessments (TN + FP). Accuracy was defined as the ratio of the number of all correct assessments (TP + TN) to the number of all assessments (TP + TN + FN + FP). Invalid and inconclusive test results were excluded from performance calculations. The 95% confidence interval (CI) was calculated according to Agresti and Coull [35].

The comparative analysis of the performance characteristics of T-Track^®^ TB and QFT-Plus was performed on samples with valid results by both methods using the McNemar test with Edwards’ correction. 

Agreement analyses were conducted between the T-Track^®^ TB assays and the QFT-Plus ELISA used as a comparative method. Positive percent agreement (PPA), negative percent agreement (NPA), and overall percent agreement (OPA) were calculated. The 95% CI values were computed according to Agresti and Coull [35]. Cohen’s kappa coefficient was used to measure the degree of agreement between the two methods; values of 0.00–0.20, 0.21–0.40, 0.41–0.60, 0.61–0.80, and 0.81–1.00 were interpreted as poor, mild, moderate, substantial, and near-perfect agreement, respectively.

Pearson’s correlation coefficient was used to evaluate quantitative correlations. Pearson’s r values < 0.2, 0.2–0.39, 0.4–0.59, 0.6–0.79, and >0.8 were interpreted as very low, low, moderate, high, and very high correlations, respectively.

## 3. Results

### 3.1. Study Participants’ Characteristics

**Figure 2:**
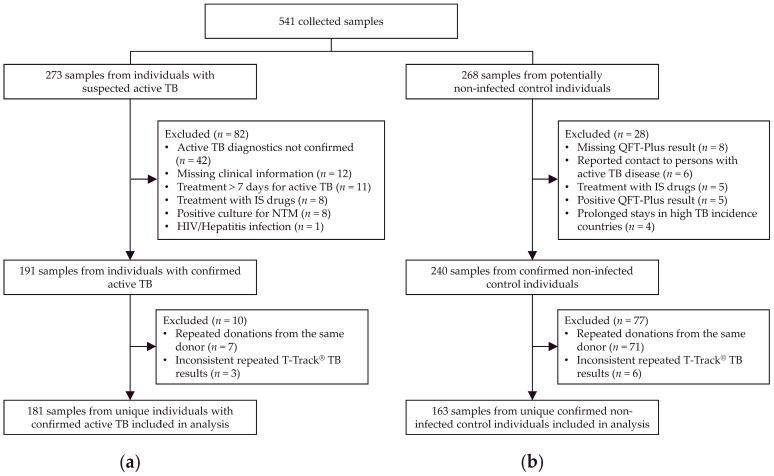
Study flow diagram. Out of 541 collected samples, 273 were from patients with suspected active TB (**a**) and 268 from presumed non-infected individuals (**b**). After exclusion of samples not meeting inclusion/exclusion criteria, and exclusion of samples from multiple collections from the same donor and with inconsistent T-Track^®^ TB results upon repeated testing, a total of 344 samples were analyzed, including 181 from patients with confirmed active TB (**a**) and 163 from confirmed non-infected controls (**b**). Abbreviations: HIV, human immunodeficiency virus; IGRA, interferon-gamma release assay; IS, immunosuppressive; MTC *Mycobacterium tuberculosis* complex; NTM, nontuberculous mycobacteria; QFT-Plus, QuantiFERON^®^-TB Gold Plus; TB, tuberculosis.

A total of 541 samples were collected, including 273 from patients with suspected active TB and 268 from presumed non-infected control individuals (Figure 2). Of these, 344 samples from unique individuals, including 181 from patients with confirmed active TB (Figure 2a) and 163 confirmed non-TB controls (Figure 2b), were included in the analysis.

The characteristics of the study participants included in the analysis are shown in Table 1. The median (range) age of TB patients and controls was 35 (18–91) and 28 (18–78) years, respectively. The group of TB patients was ethnically more diverse (52.5% European, 22.6% African, 22.1% Asian) than the control group (99.4% European) and included more men (69.1% vs. 36.2% in TB patients vs. controls) (Table 1). Of 181 analyzed TB patients, 177 (97.8%) were defined as active TB by a positive *Mtb* culture and four (2.2%) by a positive microscopy and PCR (Table S2). In addition, 153/181 (84.5%) TB patients were diagnosed with pulmonary TB, either as single-organ involvement (122/181 [67.4%]) or with extra-pulmonary organ involvement (31/181 [17.1%]), while 28/181 (15.5%) were diagnosed with extra-pulmonary TB only (Table S2).

Out of the 344 T-Track^®^ TB tests performed, 311 (90.4%) were based on results for both markers, 29 (8.4%) on a result for IFNG mRNA alone, none (0.0%) on a result for CXCL10 mRNA alone, and four (1.2%) were invalid (Figure S1).

### 3.2. Diagnostic Performance of T-Track^®^ TB

Out of 181 samples from active TB patients, three with invalid and one with inconclusive T-Track^®^ TB results were excluded from the performance analysis. Out of 163 samples from control individuals, one with invalid and one with inconclusive T-Track^®^ TB results were excluded from the performance evaluation. Thus, 338 samples were included in the performance evaluation (Figure S1).

**Figure 3:**
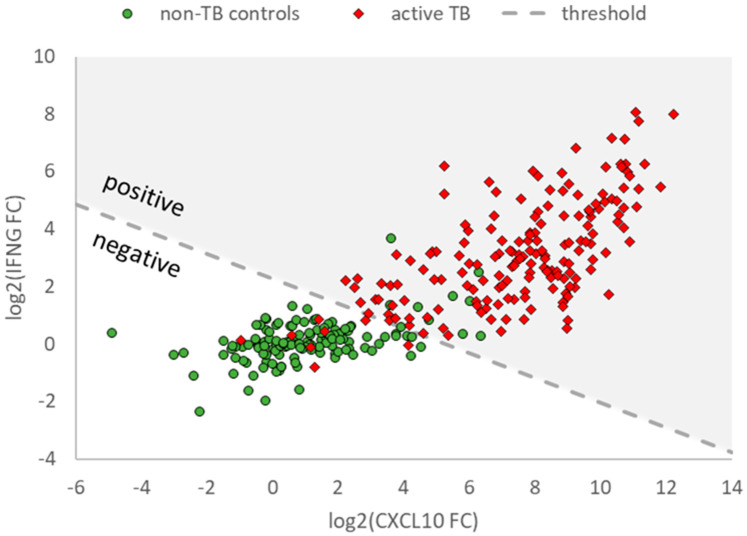
*IFNG* and *CXCL10* mRNA expression levels in samples from patients with active TB and uninfected study participants obtained with T-Track^®^ TB. Scatter plot showing valid log2-transformed fold change (FC) values of *IFNG* and *CXCL10* mRNA levels in samples from patients with active TB (red diamonds) and uninfected study participants (green circles). Only samples with valid results for both markers are shown (*n* = 176 active TB, *n* = 135 non-TB controls). The dashed line (classification threshold) classifies the samples into “positive” (upper right) and “negative” (lower left).

Out of these 338 samples, 311 (176 in active TB patients and 135 in non-TB controls) had valid fold change (FC) results for both *IFNG* and *CXCL10* (Figure S1). Changes in mRNA levels of *IFNG* and *CXCL10* mRNA (log2-transformed *IFNG* and *CXCL10* FC values) in these samples are represented in Figure 3 to illustrate the discrimination of active TB (red diamonds) and non-TB controls (green circles) relative to the classification threshold. 

The diagnostic performance of T-Track^®^ TB to discriminate active TB patients from non-TB controls in the 338 evaluated valid samples (177 active TB and 161 non-TB controls; Figure S1) is shown in Table 2. Overall, 168/177 active TB samples were correctly classified as positive and 151/161 controls samples were correctly classified as negative, corresponding to sensitivity, specificity, and accuracy (95% CI) values for T-Track^®^ TB of 94.9% (90.5–97.4%), 93.8% (88.8–96.7%), and 94.4% (91.3–96.4%), respectively (Table 2). 

The rate of invalid and inconclusive T-Track^®^ TB results in the 344 evaluated samples was 1.16% and 0.58%, respectively (Table 2).

### 3.3. Comparison of T-Track^®^ TB and QFT-Plus Test Results

#### 3.3.1. Qualitative Assay Performance Comparison

The sensitivity of the QFT-Plus assay was evaluated on the same TB samples (*n* = 181). Two samples with missing QFT-Plus test results and one indeterminate QFT-Plus result were excluded from the calculation (Figure S2). Overall, 150/178 evaluated active TB cases were correctly classified as positive by QFT-Plus, corresponding to sensitivity (95% CI) of 84.3% (78.2–88.9) (Table 2). 

The difference in sensitivity between T-Track^®^ TB and QFT-Plus tested on common samples (*n* = 174; Figure S3) was statistically significant (94.8% vs. 85.1%; McNemar, *p* < 0.001). 

A subgroup sensitivity analysis was conducted on sputum-negative and sputum-positive pulmonary TB (Table S3). Of 153 pulmonary TB patients (Table S2), 146 (95.4%) had a documented sputum status. While the sensitivity of T-Track^®^ TB to detect active TB in sputum-negative and sputum-positive samples was comparably high in both groups (93.0% vs. 95.3%; Fisher’s exact test, *p* = 0.714), the sensitivity of QFT-Plus appeared lower in sputum-negative samples (78.6%) compared to sputum-positive samples (86.4%), although the difference was not statistically significant (Fisher’s exact test, *p* = 0.255) (Table S3). As for the total TB population, the difference in sensitivity between T-Track^®^ TB and QFT-Plus tested on common samples within the sputum-negative (*n* = 55) and sputum-positive (*n* = 85) population was statistically significant (92.7% vs. 80.0% for sputum-negative patients and 95.3% vs. 87.1% for sputum-positive patients; McNemar, *p* = 0.046 in both cases) (Table S3). 

The specificity and accuracy of QFT-Plus could not be calculated (Table 2) because a QFT-Plus-negative result was an inclusion criterion for the non-infected control group (Figure 1). The rate of invalid and inconclusive QFT-Plus results in the 342 evaluated samples was 0.00% and 0.29%, respectively (Table 2). 

#### 3.3.2. Qualitative Assay Concordance

A concordance analysis of T-Track^®^ TB with QFT-Plus results was performed on 174 active TB samples with valid qualitative test results in both assays (Figure S3). Overall, 153/174 test results were concordant (146 positives, 7 negatives), corresponding to an overall agreement rate of 87.9% (Table 3). Moreover, 146/148 positive QFT-Plus were Track^®^ TB-positive, corresponding to a positive agreement rate of T-Track^®^ TB with QFT-Plus of 98.7% (Table 3). Only 7/26 negative QFT-Plus results were also Track^®^ TB-negative, corresponding to a negative agreement rate of T-Track^®^ TB with QFT-Plus of 26.9% (Table 3). Out of the 21 samples with discordant results, 19 were correctly classified by T-Track^®^ TB while misclassified by QFT-Plus, and two were misclassified by T-Track^®^ TB while correctly classified by QFT-Plus (Table 3). These two QFT-Plus-positive, T-Track^®^ TB-negative samples showed low TB1-Nil and TB2-Nil results of 0.42/0.65 IU/mL and 0.45/0.76 IU/mL, respectively.

**Figure 4:**
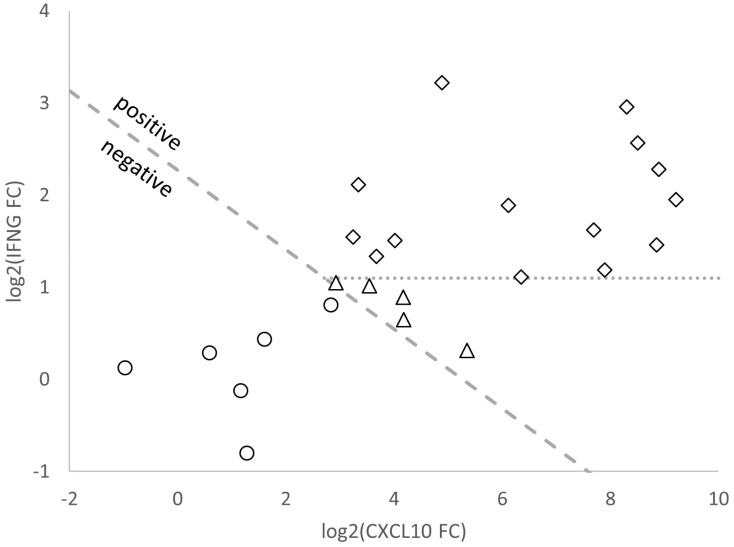
Scatter plot of log2 fold change (FC) values of *CXCL10* and *IFNG* mRNA expression generated by T-Track^®^ TB from samples of 25 TB patients classified as false negative by QFT-Plus. Samples shown with white circles were also misclassified by T-Track^®^ TB (rated as negative by both assays). Samples shown with diamonds and triangles were correctly classified based on log2-transformed FC values of *IFNG* and *CXCL10* mRNA markers by T-Track^®^ TB. Samples shown with diamonds above the dashed line were also classified as positive by the determination of the *IFNG* mRNA marker alone, as opposed to the samples shown as triangles below the dashed line. This indicates the benefit of combining *IFNG* and *CXCL10* mRNA FC results in T-Track^®^ TB to improve assay sensitivity.

Out of the 26 negative QFT-Plus test results (i.e., active TB patients misclassified as non-infected by QFT-Plus; Table 3), 25 had valid T-Track^®^ TB results for both *CXCL10* and *IFNG* markers, and one had no valid T-Track^®^ TB result for *CXCL10* and was only rated by *IFNG* single-marker analysis. A representation of the log2 FC values of *CXCL10* and *IFNG* mRNA levels determined by T-Track^®^ TB for these 25 samples (19 true positives and 6 false negatives) is shown in Figure 4. The six samples that were false negative with both assays are depicted as empty circles (below the positivity threshold). Out of the 19 true positive samples (above the positivity threshold; Figure 4), 14 (73.7%) would have been rated as positive based on the *IFNG* mRNA FC result alone (empty diamonds above the horizontal threshold for *IFNG* positivity; Figure 4), while five (26.3%) would have been missed without the consideration of *CXCL10* mRNA FC in the T-Track^®^ TB test evaluation (empty triangles below the horizontal dotted line). 

#### 3.3.3. Quantitative Assay Correlation

**Figure 5:**
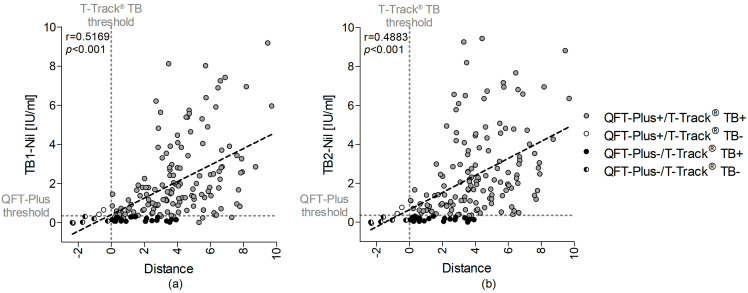
Correlation analysis between QFT-Plus results for (**a**) TB1-Nil or (**b**) TB2-Nil (IU/mL) and T-Track^®^ TB distance values for 164 donors with active TB. The 19 TB patients misclassified by QFT-Plus are shown as black circles, the 2 patients misclassified by T-Track^®^ TB are depicted as white circles and the 6 patients falsely classified by both tests in black–white circles. TB patients correctly classified with both tests are depicted in grey. The vertical dashed line depicts the T-Track^®^ TB classification threshold defining the T-Track^®^ TB assay result (“positive” vs. “negative”), and the horizontal dashed line represents the QFT-Plus threshold. Regression analysis was performed by linear correlation. QFT-Plus+/−, positive/negative QuantiFERON^®^-TB Gold Plus test result; T-Track TB+/−, positive/negative T-Track^®^ TB test result.

The correlation between T-Track^®^ TB distance from threshold values and quantitative QFT-Plus results for TB1-Nil and TB2-Nil (IU/mL) was evaluated in 164 active TB patients with complete quantitative results for all markers in both assays (Figure 5). Data from 10 donors were excluded from this evaluation because of missing quantitative QFT-Plus results (nine samples) or a missing distance result for T-Track^®^ TB (due to one invalid *CXCL10* result) (Figure S3). There was a moderate correlation between the distance values of T-Track^®^ TB and the TB1-Nil (r = 0.517, *p* < 0.001) and TB2-Nil values (r = 0.488, *p* < 0.001) of QFT-Plus, respectively (Figure 5).

## 4. Discussions

This study reports the first evaluation of the diagnostic performance of T-Track^®^ TB, an RT-qPCR-based assay intended as an aid in the diagnosis of *Mtb* infection. T-Track^®^ TB measures *IFNG* and *CXCL10* mRNA levels from small volumes of whole blood stimulated with *Mtb*-specific ESAT-6 and CFP-10 protein antigens. T-Track^®^ TB results were compared to those of the commercial ELISA-based QuantiFERON^®^-TB Gold Plus (QFT-Plus) assay, which measures interferon-γ released by T-helper cells and cytotoxic T cells in response to specific stimulation with pools of peptides derived from the *Mtb* proteins ESAT-6 and CFP-10 [6].

T-Track^®^ TB demonstrated good performance, with sensitivity of 94.2% and specificity of 93.8% in detecting active TB vs. non-TB controls. In addition, the sensitivity of T-Track^®^ TB was significantly higher than that of QFT-Plus, both in the whole TB population (*p* < 0.001) and within the sputum-negative and sputum-positive groups (*p* = 0.046 in both cases). Interestingly, the sensitivity of T-Track^®^ TB in sputum-negative vs. sputum-positive pulmonary TB patients differed by only 2.3 percentage points (93.0% vs. 95.3%; *p* = 0.714) vs. 7.8 percentage points for QFT-Plus (78.6% vs. 86.4%; *p* = 0.255). Although the difference in sensitivity between the sputum status groups for QFT-Plus was not statistically significant, this observation suggests that T-Track^®^ TB results might be less affected by the sputum status of TB patients. It thus might be a more robust assay than the comparative QFT-Plus method. This preliminary observation should be confirmed on a larger sputum-negative vs. sputum-positive pulmonary TB population, allowing an analysis with higher statistical power. 

The sensitivity of QFT-Plus in detecting active TB patients observed in our study (84.3%) was lower than that of a recent meta-analysis reporting a pooled sensitivity of 92.6% in microbiologically confirmed active TB patients and 85.5% in clinically diagnosed active TB patients [36]. These differences may be explained by the sample handling method and the study population’s characteristics. In our study, samples were collected at different study centers in Germany and Austria and were transported within a maximum of 8 h over long distances to the measurement centers, reflecting sample shipment and processing at many healthcare centers.

The superior sensitivity of T-Track^®^ TB over QFT-Plus was further demonstrated in the agreement analysis conducted in patients with confirmed TB, which showed the strong positive agreement (98.7%) of T-Track^®^ TB with QFT-Plus and low negative agreement (26.9%), reflecting the high proportion (19/26 [73.1%]) of active TB patients misclassified by QFT-Plus but correctly classified by T-Track^®^ TB. The improved positivity rate of T-Track^®^ TB compared to QFT-Plus is probably due to the methodology employed (RT-qPCR vs. ELISA) and the combined assessment of *IFNG* and *CXCL10* mRNA biomarkers. This was demonstrated for at least 5/19 (26.3%) samples misclassified by QFT-Plus, which were correctly classified by T-Track^®^ TB thanks to the combined assessment of *IFNG* and *CXCL10* (while *IFNG* was sufficient to correctly classify 14/19 [73.7%] samples otherwise misclassified by QFT-Plus). In addition, the molecular assay readout (one-step multiplex RT-qPCR) and the optimized evaluation algorithm (T-Track^®^ TB Calculator) likely contribute to a simple, precise, and reliable interpretation of results, allowing the sensitive identification of TB patients and their discrimination from non-TB controls. 

Others recently suggested such a molecular assay design to improve the performance of currently available IGRAs. In previous studies, molecular tests based on detecting various single mRNA markers and combinations thereof were evaluated to improve TB diagnosis. Kim et al. [15] reported a 100% and 71.43% positivity rate for RT-qPCR assays evaluating single *CXCL10* and *IFNG* mRNA biomarkers, respectively, in a group of 28 patients with active pulmonary TB. The negativity rate for these single markers in a group of 29 non-TB controls was 93.10% and 96.55%, respectively [15]. Although this study did not investigate the performance when combining *CXCL10* and *IFNG* mRNA, it demonstrated the benefit of combining suitable mRNA markers, such as *IFNG*, *TNFα* and *IL-2R* or *TNFα*, *IL-2R*, *CXCL9*, and *CXCL10*, to improve TB diagnosis and differentiate *Mtb* infection status [15]. On the other hand, Blauenfeldt et al. [37] reported sensitivity and specificity for a *CXCL10* mRNA assay of 80% and 98% [25]. Recently, Pan et al. reported comparable performance for a molecular *CXCL10* mRNA assay vs. T-Spot.TB in 352 patients with definite TB and 153 non-TB controls, with sensitivity of 93.9% vs. 94.5% and specificity of 98.0% vs. 100% for the diagnosis of *Mtb* infection. However, in a subgroup of 14 TB patients with HIV co-infection, the molecular *CXCL10* mRNA test showed a significantly higher positive rate (92.9%) than T-Spot.TB (61.5%; *p* = 0.029) [37]. Varying stimulation protocols, such as distinct stimulants or stimulation times, may explain the differences in the performance of the molecular tests described in the literature. The stimulation time is a critical performance parameter for molecular assays, as mRNA markers are subject to dynamic turnover. The nature of the stimulating antigens may also impact the performance of the respective assays. Compared to the currently used IGRAs, which employ pools of peptides derived from the *Mtb* ESAT-6 and CFP-10 antigens [38], T-Track^®^ TB uses highly pure heterodimers of the *Mtb* ESAT-6 and CFP-10 proteins for the specific detection of *Mtb*-reactive T cells. ESAT-6 and its chaperone CFP-10 have been previously described to form a tight, soluble, and highly stable 1:1 complex [33,34]. The use of whole proteins instead of peptides may increase the sensitivity of the assay due to the presentation of a broader range of epitopes upon the degradation of whole proteins into peptides, as previously suggested [39], resulting in the more efficient stimulation of T cells, potentially yielding higher *IFNG* and *CXCL10* mRNA levels.

Molecular methods such as RT-qPCR represent a highly competitive technological platform to improve current T-cell-based immunological assays. The advantages of this approach are the high assay sensitivity, the possibility of the simultaneous analysis of different markers from small sample volumes using multiplex RT-qPCR, the automatability, the high sample throughput, and the short time to obtain results. In addition, these molecular assays might not be affected by pre-existing cytokines and chemokines or the number of immune cells in the blood sample because they quantify the relative mRNA levels of immune markers (in antigen-stimulated vs. unstimulated conditions) [15]. Furthermore, RT-qPCR-based diagnostic methods are now widely used in diagnostic laboratories and medical care centers.

A major strength of this study is the side-by-side comparison of both tests using aliquots of identical blood samples and using comparable stimulation conditions (transfer of blood samples to stimulation tubes immediately before the start of the incubation). The main limitation of this study is the use of active TB patients as a surrogate group for TB infection. Given the absence of a gold standard for detecting latent *Mtb* infection, we chose to perform this first proof-of-concept of the molecular platform using a case–control study design including microbiologically confirmed TB patients. To validate this platform further for TB infection diagnosis, apart from non-TB controls and active TB patients, a latent *Mtb* infection group and immunocompromised patients should be included in future assay evaluations.

## 5. Conclusions

The performance comparison of the novel T-Track^®^ TB molecular assay, measuring *IFNG* and *CXCL10* mRNA levels in whole blood stimulated with *Mtb*-specific ESAT-6 and CFP-10 proteins, with an existing ELISA-based IGRA (QFT-Plus), demonstrated the suitability of T-Track^®^ TB to accurately discriminate active TB patients from non-TB controls. 

## Figures and Tables

**Table 1:** Demographics of study participants according to TB infection status.

Characteristics	TB Patients	Controls
Study population, *N* (%)	181 (100.0%)	163 (100.0%)
Age in years, median (range)	35 (18–91)	28 (18–78)
Sex, *N* (%)		
Male	125 (69.1%)	59 (36.2%)
Female	56 (30.9%)	104 (63.8%)
Ethnicity, *N* (%)		
African	41 (22.7%)	0 (0.0%)
Latin American	2 (1.1%)	0 (0.0%)
Asian	40 (22.1%)	1 (0.6%)
European	95 (52.5%)	162 (99.4%)
Unknown	3 (1.7%)	0 (0.0%)

Abbreviation: TB, tuberculosis.

**Table 2:** Diagnostic performance of T-Track^®^ TB and QFT-Plus to discriminate active TB patients from non-infected controls.

Assay	Sensitivity n/N (%) [95% CI]	Specificity n/N (%) [95% CI]	Accuracy n/N (%) [95% CI]	Invalid Rate n/N (%)	Inconclusive Rate n/N (%)
**T-Track^®^ TB** ^1^	168/177 (94.9%) [90.5–97.4]	151/161 (93.8%) [88.8–96.7]	319/338 (94.4%) [91.3–96.4]	4/344 (1.16%)	2/344 (0.58%)
**QFT-Plus** ^2^	150/178 (84.3%) [78.2–88.9]	n.d. ^3^	n.d. ^3^	0/342 (0.00%)	1/342 (0.29%)

^1^ After exclusion of four invalid and two inconclusive T-Track^®^ TB results, performance was evaluated on 177 active TB and 161 control samples (Figure S1). ^2^ After exclusion of one indeterminate and two missing QFT-Plus results, performance was evaluated on 178 active TB and 163 control samples (Figure S2). ^3^ Specificity and accuracy of the QFT-Plus assay cannot be determined in the current study design because QFT-Plus-negative result was defined as inclusion criterion for non-infected controls. Abbreviations: CI, confidence interval; n.d., not determined; QFT-Plus, QuantiFERON^®^-TB Gold Plus; TB, tuberculosis.

**Table 3:** Concordance of T-Track^®^ TB and QFT-Plus assays on samples of active TB patients.

T-Track^®^ TB Result (*n*)	QFT-Plus Result (*n*)			Agreement % (95% CI)			Kappa Value (95% CI)
	Positive	Negative	Total	Overall Percent Agreement	Positive Percent Agreement	Negative Percent Agreement	
**Positive** **Negative** **Total**	146 2 148	19 7 26	165 9 174	87.9 (82.2–92.0)	98.7 (94.9–99.9)	26.9 (13.5–46.3)	0.35 (0.15–0.56)

Abbreviations: CI, confidence interval; QFT-Plus, QuantiFERON^®^-TB Gold Plus; TB, tuberculosis.

## Data Availability

The data presented in this study are available within the article and Supplementary Materials.

## Supplementary Materials

The following supporting information can be downloaded at: https://www.mdpi.com/article/10.3390/diagnostics13040758/s1, Table S1: Recruiting centers and number (N) of suspected active tuberculosis (TB) patients and non-TB controls enrolled in the study; Table S2: Characteristics of the active TB samples included in the analysis (*n* = 181); Table S3: Diagnostic sensitivity of T-Track^®^ TB and QFT-Plus in pulmonary TB patients, according to their sputum status (*n* = 146 pulmonary TB patients with either a sputum-negative [*n* = 58] or a sputum-positive [*n* = 88] status); Figure S1: Description of the T-Track^®^ TB test results conducted in the 344 analyzed samples (181 from active TB patients, 163 from non-TB controls); Figure S2: Description of the QuantiFeron^®^-TB Gold Plus (QFT-Plus) test results conducted in the 344 analyzed samples (181 from active TB patients, 163 from non-TB controls); Figure S3: Description of the common active TB samples with valid test results with both T-Track^®^ TB and QuantiFeron^®^-TB Gold Plus (QFT-Plus).
