# Supplementary material for: Performance of T-Track® TB, a Novel Dual Marker RT-qPCR-Based Whole-Blood Test for Improved Detection of Active Tuberculosis
Source: Diagnostics (Basel). 2023 Feb 16;13(4):758. doi: 10.3390/diagnostics13040758 (PMC9955725; doi:10.3390/diagnostics13040758)
Supplement: Supplementary file 1 [file diagnostics-13-00758-s001.zip › Supplementary Figures S1-S3.pdf]

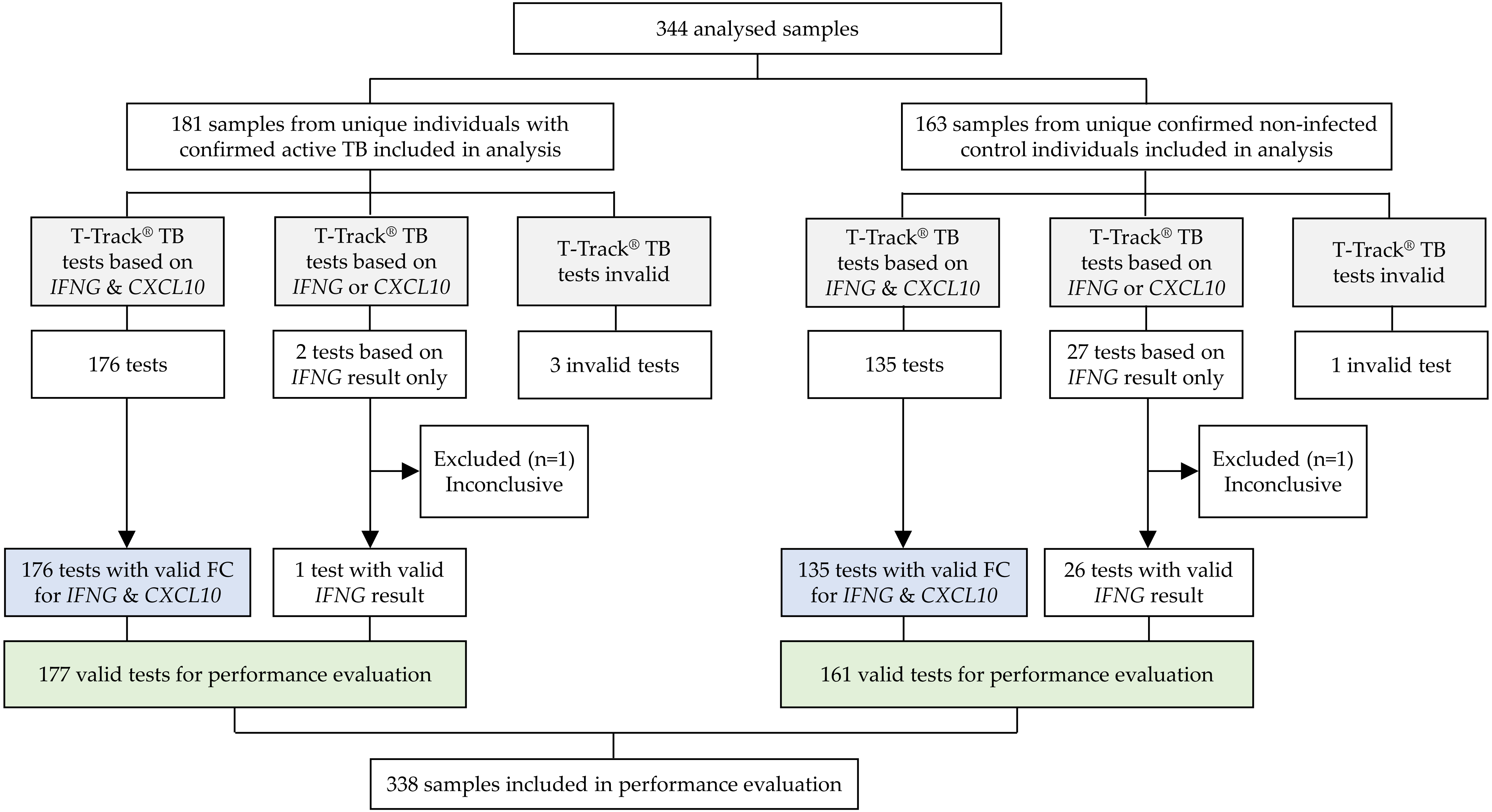

Figure S1. Description of the T-Track® TB test results conducted in the 344 analysed samples (181 from active TB patients, 163 from non-TB controls). The 338 samples included in the performance evaluation (**Table 2**) are highlighted in green (177 valid test results in the TB group, 161 valid test results in the non-TB group). The samples with valid fold-change (FC) values for both *IFNG* and *CXCL10* (176 in the TB group, 135 in the non-TB group) depicted in **Figure 3** are highlighted in blue.

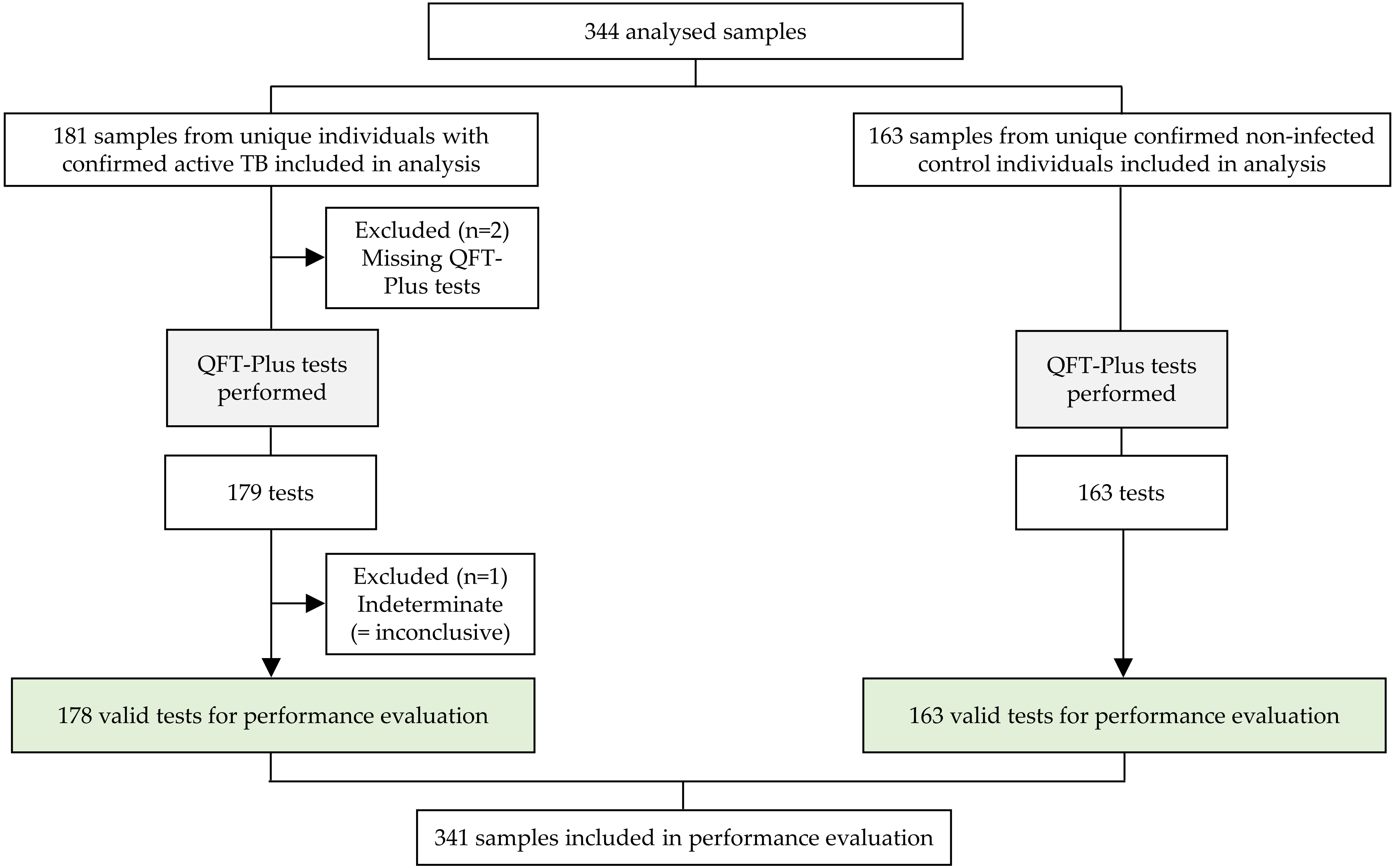

Figure S2. Description of the QuantiFeron®-TB Gold Plus (QFT-Plus) test results conducted in the 344 analysed samples (181 from active TB patients, 163 from non-TB controls). The 341 samples included in the performance evaluation (**Table 2**) are highlighted in green (178 valid test results in the TB group, 163 valid test results in the non-TB group).

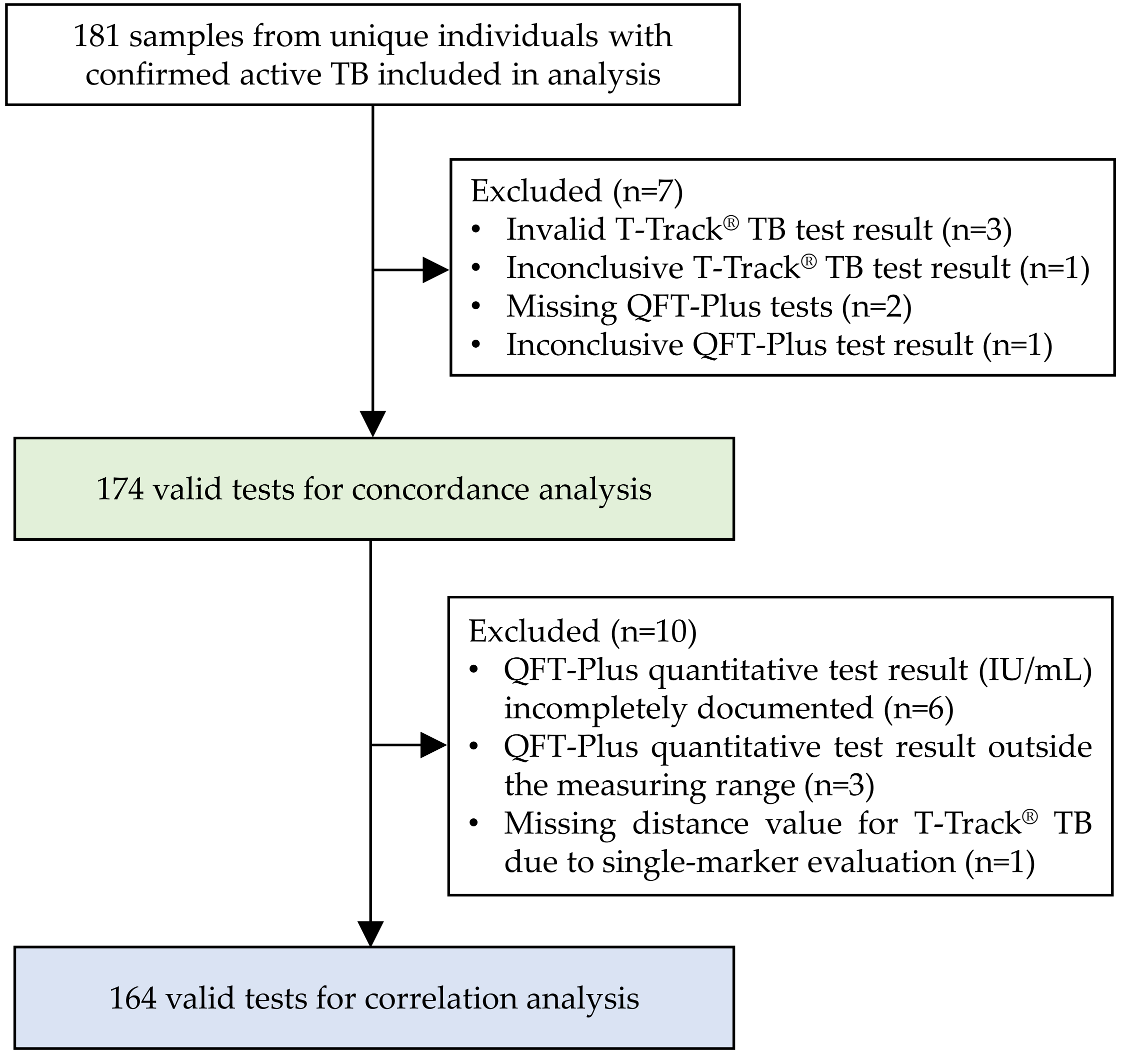

Figure S3. Description of the common active-TB samples with valid test results with both T-Track® TB and QuantiFeron®-TB Gold Plus (QFT-Plus). The 174 common samples with valid qualitative test results included in the concordance analysis (**Table 3**) are highlighted in green. The 164 common valid samples with complete quantitative test results included in the correlation analysis (**Figure 5**) are highlighted in blue.
