# Supplementary material for: Performance of T-Track® TB, a Novel Dual Marker RT-qPCR-Based Whole-Blood Test for Improved Detection of Active Tuberculosis
Source: Diagnostics (Basel). 2023 Feb 16;13(4):758. doi: 10.3390/diagnostics13040758 (PMC9955725; doi:10.3390/diagnostics13040758)
Supplement: Supplementary file 1 [file diagnostics-13-00758-s001.zip › Supplementary Tables S1-S3.pdf]

Table S1. Recruiting centres and number (N) of suspected active tuberculosis (TB) patients and non-TB controls enrolled in the study

**Study Center**	**Active TB (N)**	**Non-TB controls (N)**	**Total (N)**
Institute for Medical Microbiology and Hygiene, University of Regensburg, Germany	0	241	241
Department of Tuberculosis/Infectious Lung Disease, Asklepios Lungenklinik Gauting, Germany	162	8	170
Institute for Transfusion Medicine, Laboratory Medicine and Medical Microbiology, Medical Center Dortmund, Germany [1]	24	11	35
Fachklinik für Pneumologie, Thoraxchirurgie, Rehabilitation, Schlaf- und Beatmungsmedizin, Münnerstadt, Germany	27	0	27
Department of Hematology, Medical Oncology, and Pneumology, University Medical Center of the Johannes Gutenberg University, Mainz, Germany	15	0	15
Center for Pneumology, Donaustauf Hospital, Germany	12	0	12
Lungenärzte am Rundfunkplatz, Munich, Germany	10	0	10
Karl Landsteiner Privatuniversität für Gesundheitswissenschaften, Universitätsklinikum Krems, Krems an der Donau, Austria	9	0	9
Klinikum Chemnitz gGmbH, Chemnitz, Germany	1	6	7
Department of Infectious Diseases and Tropical Medicine, Klinikum St. Georg gGmbH Leipzig, Germany	4	2	6
Klinikum Wels-Grieskirchen GmbH, Wels, Austria	4	0	4
Department for Pneumology and Critical Care Medicine, Thoraxklinik University of Heidelberg, Germany	2	0	2
Internal Medicine 2, University Hospital of St. Pölten, Sankt Pölten, Austria	2	0	2
Department of Internal Medicine I, University Clinical Centre Tübingen, Germany	1	0	1
**Total (N)**	273	268	541

[1] Also acting as one of the two measuring centers (together with Mikrogen GmbH, Regensburg, Germany)

Table S2. Characteristics of the active TB samples included in the analysis (n=181)

**Characteristics**	**N (%)**
Study population	181 (100.0%)
Active TB diagnosis	
Positive *Mtb* culture [1]	177 (97.8%)
Positive microscopy and PCR	4 (2.2%)
Organ involvement	
Pulmonary TB	122 (67.4%)
Extra-pulmonary TB	28 (15.5%)
Pulmonary and extra-pulmonary TB	31 (17.1%)

[1] Of the 177 culture-positive analysed patients, 92 (52.0%) were simultaneously microscopy- and PCR-positive for TB. Abbreviations: *Mtb*, *Mycobacterium tuberculosis*; TB, tuberculosis.

Table S3. Diagnostic sensitivity of T-Track® TB and QFT-Plus in pulmonary TB patients, according to their sputum status (n=146 pulmonary TB patients with either a sputum-negative [n=58] or a sputum-positive [n=88] status)

**Assay**	**Sputum status**	**Sensitivity**	
		**n/N (%)**	**[95% CI]**
**T-Track® TB** [1]	Negative	53/57 (93.0%)	[82.8–97.7]
	Positive	81/85 (95.3%)	[88.1–98.5]
**QFT-Plus** [2]	Negative	44/56 (78.6%)	[66.0–87.4]
	Positive	76/88 (86.4%)	[77.5–92.2]

[1] After exclusion of three invalid and one inconclusive T-Track® TB results, sensitivity was evaluated on 142 pulmonary TB samples (57 sputum-negative, 85 sputum-positive); [2] After exclusion of one indeterminate and one missing QFT-Plus results, sensitivity was evaluated on 144 pulmonary TB samples (56 sputum-negative, 88 sputum-positive). A Fisher's exact test showed no statistically significant difference between the sensitivity of sputum-negative and sputum-positive detection by T-Track® TB (p=0.714) or by QFT-Plus (p=0.255). As for the total TB population (Table 2 and section 3.3.1.), the difference in sensitivity between T-Track® TB and QFT-Plus, tested on common samples within the sputum-negative (n=55) and sputum-positive (n=85) population, was statistically significant (92.7% *vs.* 80.0% for sputum-negative patients and 95.3% *vs.* 87.1% for sputum-positive patients; McNemar, p=0.046 in both cases). Abbreviations: CI, confidence interval; QFT-Plus, QuantiFERON®-TB Gold Plus; TB, tuberculosis.
